# Differential prioritization of therapies to subtypes of triple negative breast cancer using a systems medicine method

**DOI:** 10.18632/oncotarget.21669

**Published:** 2017-10-09

**Authors:** Henri Wathieu, Naiem T. Issa, Aileen I. Fernandez, Manisha Mohandoss, Deanna M. Tiek, Jennifer L. Franke, Stephen W. Byers, Rebecca B. Riggins, Sivanesan Dakshanamurthy

**Affiliations:** ^1^ Georgetown-Lombardi Comprehensive Cancer Center, Department of Oncology, Georgetown University Medical Center, Washington, DC, 20057 USA; ^2^ Department of Biochemistry and Molecular Biology, Georgetown University, Washington, DC, 20057 USA

**Keywords:** triple negative breast cancer, systems biology, gene expression analysis, molecular subtyping, drug development

## Abstract

Triple negative breast cancer (TNBC) is a group of cancers whose heterogeneity and shortage of effective drug therapies has prompted efforts to divide these cancers into molecular subtypes. Our computational platform, entitled GenEx-TNBC, applies concepts in systems biology and polypharmacology to prioritize thousands of approved and experimental drugs for therapeutic potential against each molecular subtype of TNBC. Using patient-based and cell line-based gene expression data, we constructed networks to describe the biological perturbation associated with each TNBC subtype at multiple levels of biological action. These networks were analyzed for statistical coincidence with drug action networks stemming from known drug-protein targets, while accounting for the direction of disease modulation for coinciding entities. GenEx-TNBC successfully designated drugs, and drug classes, that were previously shown to be broadly effective or subtype-specific against TNBC, as well as novel agents. We further performed biological validation of the platform by testing the relative sensitivities of three cell lines, representing three distinct TNBC subtypes, to several small molecules according to the degree of predicted biological coincidence with each subtype. GenEx-TNBC is the first computational platform to associate drugs to diseases based on inverse relationships with multi-scale disease mechanisms mapped from global gene expression of a disease. This method may be useful for directing current efforts in preclinical drug development surrounding TNBC, and may offer insights into the targetable mechanisms of each TNBC subtype.

## INTRODUCTION

Breast cancer is classified into multiple molecular subtypes that have been studied in association with a range of biological and clinical features, including tumor initiation, maintenance, progression, metastasis, and response to therapy. Of the major established subtypes based on hormone and growth factor receptor expression [1], triple negative breast cancer (TNBC) represents an aggressive subtype with the worst prognosis [2]. Due to lack of classical hormone receptor expression (ER, PR) or HER2 amplification, targeted therapies directed at these receptors are ineffective for TNBC, and current treatment options rely on traditional chemotherapeutics, radiotherapy and surgery [3]. Understanding the molecular features underpinning TNBC is a critical unmet need for drug discovery. Lehmann *et al* performed a seminal molecular subtyping study of TNBC using an aggregation of public mRNA expression datasets, identifying six subtypes which include two basal-like subtypes (basal-like 1 and basal-like 2), a luminal androgen receptor subtype, an immunomodulatory subtype, and two mesenchymal-like subtypes (mesenchymal-like and mesenchymal stem-like) [4]. The gene expression-based identification of TNBC subtypes has been confirmed and extended since the original study by Lehmann *et al* [5–8], and these TNBC subtypes exhibit reproducible and actionable differences in categories such as ontology, pathological complete response (pCR) in the clinic, and drug sensitivities [9–11].

Computational methods in drug development have yet to effectively integrate molecular subtyping with targeted approaches to therapy. Targeted therapies have transformed the treatment of receptor-positive breast cancers and many other tumors types, such as papillary thymic carcinoma treated with kinase inhibitors [12, 13]. However, the success of most targeted therapies is predicated upon the patient's tumor exhibiting a specific targetable genomic lesion, for example V600E B-Raf in melanoma, or ERBB2 (HER2) gene amplification in breast cancer, both of which lead to increased downstream signaling and tumorigenic activity. An alternative approach is to target one or more pathways that are aberrantly activated downstream of the genomic lesion, e.g. PI3K [14, 15]. This strategy relies on large-scale omics technologies, predominantly gene expression data, to characterize a disease more globally. The Connectivity Map (CMap) is one well-established holistic method [16–18] based on the principle of “inverse associations,” where drugs that induce gene expression changes that are in opposition to those of a specific disease state are more strongly predicted to have therapeutic benefit. By applying this principle of “inverse associations” to targeted therapy in the context of TNBC, a computational drug development platform may overcome its heterogeneity and the difficulty of identifying suitable drug targets for this aggressive breast cancer subtype.

Here, we present a novel drug discovery and repurposing platform entitled GenEx-TNBC that capitalizes on TNBC subtyping modeled after Lehmann *et al*. Our platform uses gene expression data for six different TNBC subtypes, drawn from both TNBC clinical specimens and established cell lines, and finds drugs with the greatest capability to simultaneously *i*. inhibit the overrepresented biological components and *ii*. activate the underrepresented biological components. This is done by harnessing the experimentally determined drug-protein target association space from publically accessible databases, and expanding this space to higher order biological entities that map onto our disease models. Specifically, drug-TNBC subtype inverse associations are performed using systems biology analytics at four biologically important tiers that include direct gene products (proteins), protein-protein interactions, pathways, and molecular functions. To our knowledge, GenEx-TNBC is the first drug discovery platform of its kind that performs global inverse associations at multiple biological tiers. GenEx-TNBC was subsequently validated by its ability to predict the efficacy of known and novel drugs for different TNBC molecular subtypes.

## RESULTS AND DISCUSSION

**Figure 1:**
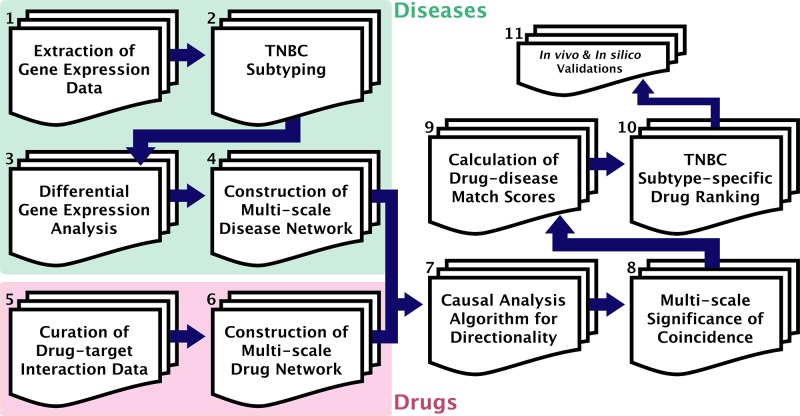
GenEx-TNBC workflow **(1)** Gene expression data obtained for cell line (Neve *et al* [19]) and patient-based (TCGA [20]) TNBC samples. **(2)** Molecular subtyping of TNBC samples performed for cell line (Lehmann *et al* [4]) and patient-based (TNBCtype [21]) data. **(3)** Significantly differentially up-regulated and down-regulated genes found for a given subtype, compared to samples of all other subtypes. **(4)** Multi-scale disease perturbation signature created by overrepresentation analysis of up- and down-regulated gene sets, separately, for associated functions (DAVID [22]) and pathways (ConsensuspathDB [23]). **(5)** Drug-protein target binding interaction data curated for FDA-approved and experimental drugs from DGIdb [24] and CTD [25]. **(6)** Multi-scale drug action signature created by annotation of pathways and functions associated with drug targets. **(7)** Directionality algorithm implemented to match drug action network to disease perturbation network based on opposite directional effect on biological entities, and irrespective of directionality at the level of targeted PPIs (STRING [26]) of disease-modulated gene products. **(8)** Hypergeometric test used to calculate statistical significance of resulting coincidence between drug and disease networks, separately at each scale of biological action. **(9)** Significance values normalized, log-transformed, and summated with weights for each level of biological action to produce drug-TNBC subtype association. **(10)** Drugs ranked against a given TNBC subtype based on descending drug-disease association score. **(11)** GenEx-TNBC findings validated based on literature findings and cell viability testing for drugs of interest against a subtyped cell line model.

**Figure 2:**
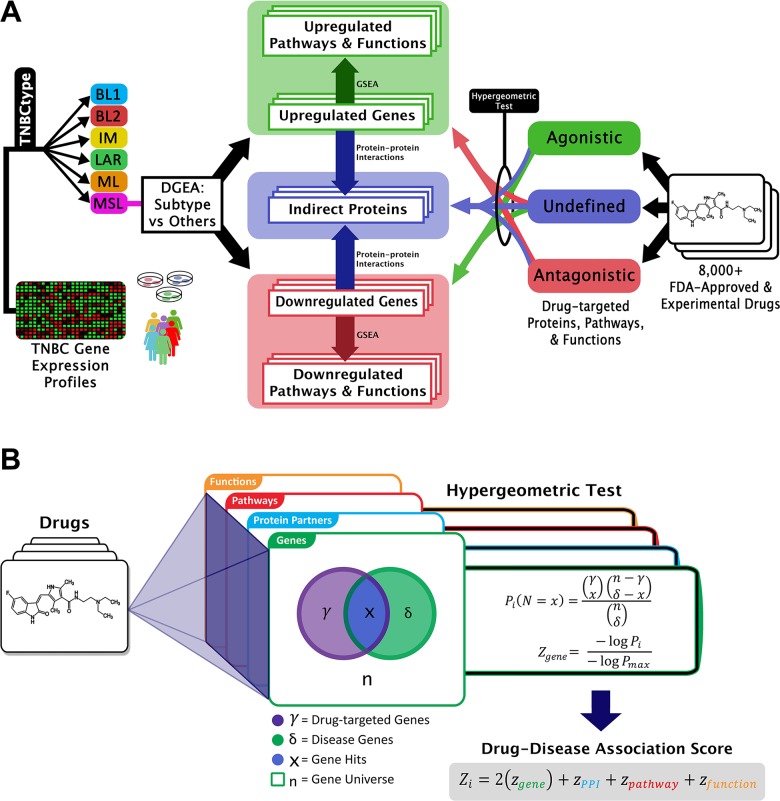
Graphical representation of GenEx-TNBC **(A)** From the left side, patient or cell line TNBC gene expression data are subtyped and differentially expressed genes are found for one subtype relative to all other subtypes. Up- and down-regulated genes are separately enriched for subtype-associated pathways and functions, and connected to indirect proteins interacting with those gene products. Drug-protein interactions with a known directional effect are matched to oppositely-regulated genes, pathways, and functions, while those with unknown effect are matched to disease-associated indirect proteins and biological entities regulated in either direction. **(B)** The hypergeometric test uses a hypergeometric distribution to determine the statistical significance of having *x* drug hits in a sample of *δ* subtype-associated biological entities. The legend describes the example case of gene level coincidence. Log-transformed p-values for each level of biological activity are summated to produce a drug-disease association score.

The GenEx-TNBC platform was used to create a prioritization rank list of 8,020 FDA-approved and experimental drugs against each of six TNBC subtypes. Following a computational workflow that serves to build drug and disease biological integrative networks (Figure 1), each drug was given an overall Z-score that was used for ranking. For a given subtype, the drug with the highest Z-score was ranked first and predicted very likely to be efficacious, and the drug with the lowest Z-score was ranked last and predicted to have the least therapeutic likelihood. GenEx-TNBC was applied using disease networks built from both TNBC patient (The Cancer Genome Atlas (TCGA) [20]) and cell line subtyped gene expression data (Neve *et al*, [19], Figure 2). This allows us to assess the extent to which cell line models might recapitulate our patient-based drug prioritization, and serves as a rationale for pursuing cell line-based biological validation of select predictions of our model. In assessing the distribution of annotated drugs, we considered the Top 100 drug-ranked for each subtype (Supplementary Figures 1, 2). Some drugs are predicted to be subtype-specific, while others are predicted to be broadly effective in multiple TNBC subtypes (Supplementary Table 1). Many of these drugs have been tested clinically in TNBC globally, as evident from the literature (Supplementary Table 2), while many others are novel candidates that warrant future preclinical, and ultimately clinical, study.

### Application of GenEx-TNBC to TCGA data

#### Basal-like subtypes

**Figure 3:**
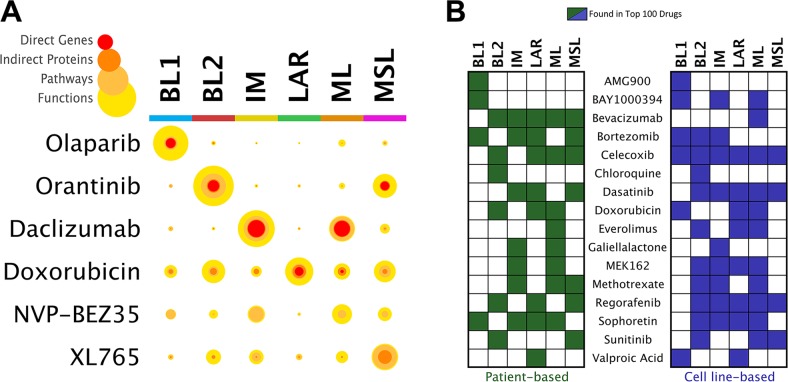
Prioritization of drugs by TNBC subtype **(A)** Statistical confidence coverage for selected drugs and TNBC subtype analyses. Color scale represents the hierarchical nature of mapped drug-subtype associations from the level of direct genes to associated proteins, pathways, and functions. For a given drug-disease pair, the width of each colored ring is proportional to the significance (hypergeometric test) to which the biological signature of the drug coincides with the patient-derived TNBC subtype signature. Each circle therefore designates the multi-scale statistical “coverage” of coincidence between the drug and TNBC subtype. **(B)** Prioritization of drugs that are of clinical interest for the treatment of TNBC, comparing analyses of disease networks deriving from patient and cell line gene expression data.

**Figure 4:**
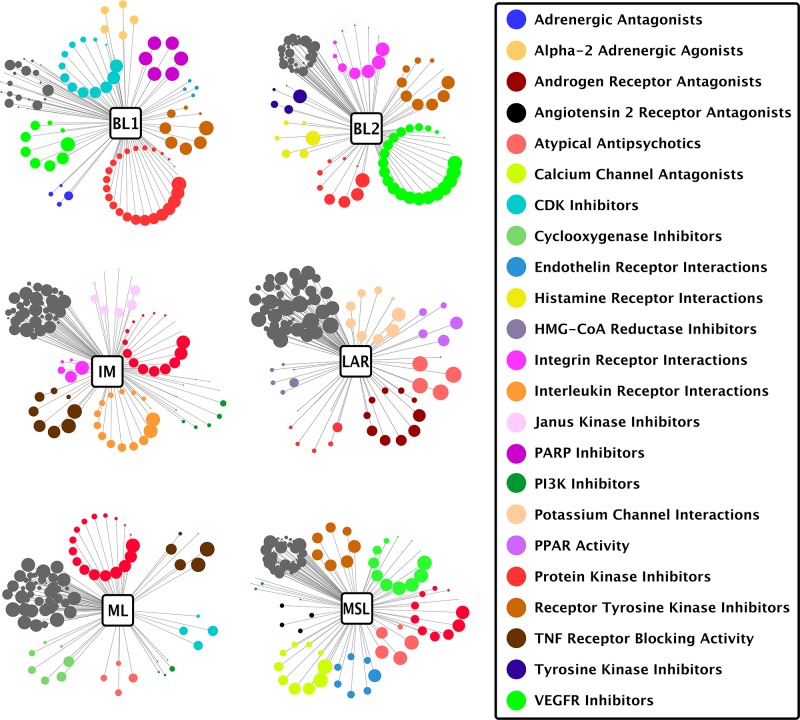
Drug classes matched to TNBC subtypes Each circle represents one drug from the top 100 drugs ranked against the subtype shown, wherein the diameter of the circle corresponds to rank position (better-ranked drugs have larger diameters) and the color of the circle indicates a drug class listed in the legend on the right. Drug classes with at least 4 drugs in the top 100 are depicted radially. Dark grey non-radial clusters represent drugs with classes not highlighted for the given subtype, and may contain drug classes listed in the legend. Drug class data were derived from the FDA NDC directory and by manual designation using GenEx-TNBC drug target profiles.

The basal-like 1 (BL1) and basal-like 2 (BL2) subtypes, both of a basal-like nature, exhibit distinct gene ontologies. From our patient-based analysis we confirmed previous findings [4] that in the BL1 subtype genes relating to the cell cycle and cell division, proliferation, and DNA damage response pathways and functions are significantly overrepresented (Supplementary Table 3). Essential to these functions are the Aurora kinases, which serve primarily to regulate chromatid segregation during mitosis. Aurora kinase inhibitors such as Alisertib, AMG900, AZD1152, and PF-03814735, were uniquely found to be in the top 100 drugs assigned to the BL1 subtype (Figures 3A, 4). Inhibitors of the poly (ADP-ribose) polymerase (PARP) family of enzymes were also predicted to target BL1 TNBCs (Figure 4), consistent with the dysfunction of DNA damage repair mechanisms associated with this subtype [27]. The BL2 subtype of TNBC is characterized by the overexpression of genes involved in growth factor signaling, glycolysis, and gluconeogenesis [4]. We specifically found angiogenic factors to be highly enriched in the BL2 subtype, and therefore inhibitors of vascular endothelial growth factor receptors (VEGFRs), platelet-derived growth factor receptors (PDGFRs), and fibroblast growth factor receptors (FGFRs) were prioritized as top drugs against BL2 (Figure 4). Orantinib, an anti-angiogenic agent that targets all three of the aforementioned receptors, has shown prior efficacy in combination with docetaxel in patients with anthracycline-resistant metastatic breast cancer [28]. GenEx-TNBC predicted Orantinib to be strongly associated with and highly specific to the BL2 subtype (Figure 3A). We also found the integrin family of cell adhesion receptors, important to both the angiogenesis and metastatic progression of solid tumors, to be both differentially expressed and having pathways overrepresented in the gene set corresponding to the BL2 subtype (Supplementary Table 3). This feature of the BL2 network produced statistical coincidence with drugs interacting with integrins, such as vitaxin and cilengitide (Figure 4), both of which have been investigated as anti-angiogenic agents against breast cancers [29]. The prominence of metastatic and angiogenic mechanisms is in accordance with previous findings that the BL2 subtype exhibits the lowest pCR following standard neoadjuvant chemotherapy (0%), even when correcting for multiple clinical factors [9].

#### Immunomodulatory subtype

The immunomodulatory (IM) subtype of TNBC expresses a robust profile of immune cell responses, a fact which has brought into question whether this activity derives from immune cell infiltrates, the tumor cells themselves, or both – in response to this, some modifications of the TNBC molecular classification schema now exclude the IM subtype [4, 5, 7]. Regardless of the precise source of immune activation, immunological infiltration is an important pathological indicator in TNBC, being suggestive of high tumor grade but relatively good prognosis [30]. Our disease network model and subsequent drug prioritization for the IM subtype may serve to illuminate ways in which modification of the immunological microenvironment in TNBC can improve drug sensitivities or pathological response [31, 32]. GenEx-TNBC prioritized drug classes that reflect two distinct approaches in cancer immunotherapy: targeting immune inhibitory checkpoints such as programmed cell death protein 1 (PD-1) or cytotoxic T-lymphocyte-associated protein 4 (CTLA-4) in order to attenuate cancer evasion; or targeting macrophages themselves to reduce angiogenesis-promoting cytokine secretion and promote immunosurveillance [33, 34]. Indeed, drugs belonging to both categories were uniquely prioritized in IM by GenEx-TNBC. CLTA-4 is a T-cell inhibitory receptor whose blockage may serve as a viable immunotherapeutic strategy against non-immunogenic breast cancers [35]. Our model calculated Belatacept, a CTLA-4 inhibitor, to be significantly associated with the IM subtype at multiple levels of biological activity. Drugs interacting with cytokine receptors were also prevalent in the IM subtype rank list (Figure 4), a therapeutic approach that has garnered attention in TNBC [36]. The anti-CD25 antibody daclizumab, for example, is significantly and specifically associated with IM at every level of biological activity as predicted by GenEx-TNBC (Figure 3A). Daclizumab has previously exhibited potential as a therapeutic modulator of T regulatory cell response in patients with metastatic breast cancer [37].

#### Luminal androgen receptor subtype

Despite the overall strong concordance between TNBC and basal-like PAM50 intrinsic breast cancer subtype, the luminal androgen receptor (LAR) subtype of TNBC is the only subtype that is not majority basal-like [9]. In fact, most LAR tumors express genes that typically cluster in the luminal and HER2 intrinsic subtypes, and are dominated by hormone- and growth factor receptor-regulated pathways [4], consistent with the assignment of CI-1033 (Canertinib), a pan ErbB family inhibitor, as a highly-ranked drug in this subtype. As has been established by previous groups [4, 10], we found that the androgen receptor (AR) and its associated signaling and downstream effectors are overrepresented in the LAR subtype. It has been demonstrated that LAR subtypes, in turn, are sensitive to drugs which modulate AR signaling or target AR directly [4], a fact that was recapitulated by GenEx-TNBC using network-based prioritization of the retinoid X receptor (RXR) agonist Bexarotene; RXR has been shown to inhibit AR signaling (Figure 4) [38]. Given the wide array of metabolic gene ontologies that characterize the LAR subtype [4], it is unsurprising that LAR has the widest variety of drug class distribution in our model (Figure 4).

#### Mesenchymal- and mesenchymal stem-like subtypes

The mesenchymal-like subtypes of TNBC, which include mesenchymal-like (ML) and mesenchymal stem-like (MSL), exhibit characteristics typical of the epithelial–mesenchymal transition (EMT), closely linked to aberrations in the Wnt/β-catenin pathway and augmented expression of growth factors such as transforming growth factor β (TGF-β) [39]. One critical difference between ML and MSL lies in the reduced expression of proliferation-associated genes in MSL vs. ML (Supplementary Table 3). The PI3K/AKT/mTOR pathway has been proposed as a targetable pathway in mesenchymal-like TNBC [4, 40]. Significantly, PI3K inhibitors such as Alpelisib (BYL719) and Voxtalisib (XL765) were matched to the MSL subtype by GenEx-TNBC (Figures 3A, 4), while mTOR inhibitors such as temsirolimus and the dual PI3K/mTOR inhibitors NVP-BEZ235 and PF-04691502 were prioritized in the ML subtype (Figures 3A, 4). The mTOR inhibitor everolimus exhibited multi-level significance of coincidence with mesenchymal-like TNBC disease networks, particularly in the ML subtype. This finding is validated by known effectiveness against TNBC cell models via inhibition of the PI3K/Akt pathway [41] and everolimus is currently being evaluated for clinical effectiveness in TNBC [40]. We would therefore posit that everolimus should be more effective against the subset of mesenchymal-like TNBCs.

### Application of GenEx-TNBC to TNBC cell line gene expression data from Neve *et al*

Having prioritized expected drugs for TNBC subtypes based on the biological activity uniquely regulated by that subtype in patient data, we next implemented GenEx-TNBC using TNBC cell line gene expression data [19]. We found general concordance between patient and cell line TNBC subtype analyses, confirming the applicability of our model to cell line-based predictions and supporting the use of cell proliferation testing as a means to validate GenEx-TNBC predicted drug sensitivities (Figure 3B). Because immortalized cell lines models are farther removed from an *in vivo* state than primary cell cultures, we expect GenEx-TNBC predictions to differ somewhat for clinical vs. cell line data. Moreover, the cell line approach considers a single sample as most representative of each subtype, instead of multiple tumor samples. This will necessarily skew the disease network toward features that are specific to the cell line in question. For these reasons, we expect the patient-based approach to potentially be more accurate in reflecting the true therapeutic potentials of drugs against subtypes of TNBC. Nevertheless, as is shown in Figure 3B, there are clear similarities between each approach in the relative distribution among TNBC subtypes of drugs that are of current interest for the treatment of TNBC. Drugs assigned broadly (to many subtypes) tend to be so in both patient- and cell line-based analyses, and the same is generally true for more subtype-specific drugs.

### In silico validation of drug prioritization results

Initial validation of predicted drug-TNBC subtype associations was performed using published pharmacological data from cell line viability studies obtained from Lawrence *et al*, who utilized quantitative mass spectroscopy to comprehensively characterize the TNBC proteome [8] and demonstrate drug sensitivity using ATP-dependent cell viability assays. We predicted several drugs confirmed by Lawrence *et al* to be effective against the majority of TNBC subtypes, such as staurosporine (a pan-kinase inhibitor), paclitaxel (a microtubule stabilizer), and bevacizumab (an anti-VEGF monoclonal antibody). These results highlight the concept that regardless of subtype, tumors exhibit, to an extent, a dependency on redundant pro-growth and maintenance biological processes such as protein turnover, cytoskeleton stability, and others. Furthermore, our predictions concur with experimental findings from Lawrence *et al* [8] for subtype-specific drugs, such as GSK-1120212 (Trametinib), a MEK inhibitor found to be effective against the DU4475 and MDA-MB-231 cell lines, which correlate most closely [4] to the IM and MSL subtypes, respectfully. GenEx-TNBC also found Trametinib to be in the top 100 drugs for IM and MSL subtypes (Supplementary Table 1).

There are, however, discrepancies between the cell line viability data published by Lawrence *et al* and our prediction method using patient data. Some drugs were found to be effective against TNBC subtype cell lines while our method did not predict them to be. For example, cell lines belonging to the BL1 and MSL subtypes were sensitive to methotrexate *in vitro* but our method did not predict methotrexate sensitivity for the BL1 subtype (Figure 3B). Tumor heterogeneity captured by GenEx-TNBC (but not the *in vitro* studies) may explain such a discrepancy, as could the potential of these drugs to affect non-protein entities such as DNA, which has been shown for methotrexate [42, 43]. There is potential in the current model for incorporation of drug-DNA interactions, as this mode of action can have pathway-specific and general physical affects that are potentially therapeutic. According to Lawrence *et al*, in most cases drug sensitivity was directly correlated to the level of expression of the drug's target protein [8]. This is particularly pronounced for Tretinoin (all-trans retinoic acid, ATRA) against the cell line HCC1806 (corresponding to the BL2 subtype) where drug sensitivity was strongly correlated with the expression of the ATRA target RXRB.

Our method also predicted drugs for TNBC subtypes in which the *in vitro* assays from Lawrence *et al* did not indicate drug sensitivity. For example, GenEx-TNBC predicted the BL2 subtype to be sensitive to vandetanib, but Lawrence *et al* did not find the BL2 cell lines they tested to be vandetanib responsive at sub-micromolar concentrations [8]. While at first glance this may be interpreted as a false positive, some experimental and pharmacological considerations may explain these outcomes. Lawrence *et al* employed quantitation of ATP to estimate cell viability following drug exposure, whereas our usage of gene expression-derived disease modelling may not strongly predict the level of metabolic activity. In addition, failures of our model to capture known biological effect may occur because the drug in question may have low binding affinity to the drug's intended targets. We acknowledge that major limitations in our current platform include lack of pharmacokinetic and binding affinity data. Such pharmacological data is important, as mechanism of action is highly dependent on the drug reaching its target and strength of binding to the drug targets, especially that drug-target signatures cannot be necessarily reduced to binary interactions given their complexity. The next iteration of our method will include these parameters to refine our predictions.

### Laboratory validation of drug prioritization results

While many targeted therapy approaches seek to inhibit important oncogenic drivers, these alterations may be poor drug targets. One alternative strategy is to broadly inhibit gene networks and molecular functions downstream of these oncogenic drivers, a focus of our method. Ryall *et al* have previously predicted multiple kinase dependencies for different TNBC cell lines, and found that the cell line that had the greatest dependency on resulted in the greatest inhibition [44]. It logically follows that inhibiting the downstream effectors of the dependent kinase could result in similar efficacy. The unique strength of GenEx-TNBC is that it takes into account these downstream effectors while simultaneously considering the regulator, which dictates the expression of other proteins, as a potential target.

As a proof-of-concept, we assessed the growth-inhibitory potential of four compounds in cell lines that best represent three distinct TNBC subtypes – HCC1937 (BL1), BT549 (ML) and MDA-MB-453 (LAR, Supplementary Table 1). Crystal violet assays (a proxy for cell number) were selected over luminescent or colorimetric cell viability assays that measure ATP or NAD(P)H, respectively, for multiple reasons. A number of small molecules, particularly kinase inhibitors, directly inhibit the firefly (*Photinus pyralis*) luciferase used in ATP-dependent assay readouts that are wholly independent of their effects on the target protein kinase [45]. Assay readouts that depend upon NAD(P)H, such as MTT (3-(4,5-dimethylthiazol-2-yl)-2,5-diphenyltetrazolium bromide), are designed to measure cellular metabolism, not cell proliferation. MTT is also widely reported to be subject to reduction directly by test compounds [46–48], including some of those we selected for validation.

### Mecamylamine

**Figure 5:**
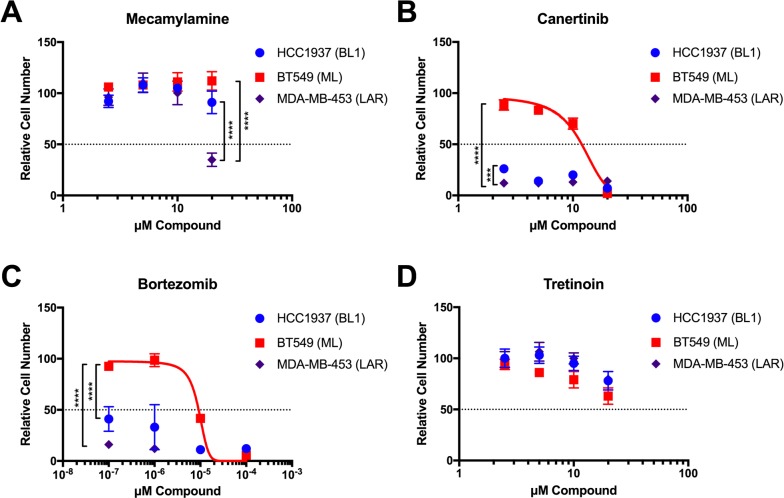
Validation of selected GenEx-TNBC predictions in TNBC cell lines of multiple molecular subtypes Crystal violet staining of HCC1937 (BL1), BT549 (ML), and MDA-MB-453 (LAR) cells grown in the presence of the indicated concentrations of Mecamylamine **(A)**, Canertinib **(B)**, Bortezomib **(C)**, and Tretinoin **(D)** for seven (7) days. Data for each cell line were normalized to the appropriate solvent control (ethanol for Mecamylamine, DMSO for all others). Data were analyzed by two-way ANOVA with Tukey *post hoc* multiple comparisons test (asterisks indicate per-dose comparisons) and are presented as the mean +/− standard deviation (S.D.) for a single experiment performed with 6 technical replicates that is representative of at least two independent experiments (biological replicates).

Mecamylamine is an orally-active, non-competitive, and non-selective nicotinic acetylcholine receptor (nAChR) antagonist originally used as an anti-hypertension drug that is also used as an anti-addictive drug for smokers [49]. GenEx- TNBC ranked Mecamylamine as 16/100 in the patient-based analysis of LAR subtype, but it is absent from the Top 100-ranked drugs for BL1 or ML subtypes. While mecamylamine has minimal activity at low doses, MDA-MB-453 (LAR) cells are significantly growth-inhibited by the highest dose tested (20 μM), while BT549 (ML) and HCC1937 (BL1) cells remain nonresponsive (Figure 5A). Several nAchRs have been implicated in oncogenesis [50], and the alpha 9 nAChR has been specifically associated with poor breast cancer-specific survival, particularly in estrogen receptor-positive (ER+) luminal breast cancer [51], potentially consistent with the luminal gene expression profile of LAR TNBC. Moreover, nAChR signaling in non-small-cell lung cancer, an epidermal growth factor receptor (EGFR)-dependent malignancy, promotes resistance to an EGFR inhibitor [52]. We therefore propose that further study of the nAChR antagonist mecamylamine in LAR TNBC is warranted, potentially in combination with an EGFR inhibitor such as Canertinib (see below).

### Canertinib

Canertinib is an orally-active, irreversible pan-ErbB family inhibitor (targets EGFR, ErbB2/HER2, HER3, and HER4) that has anti-tumor and radiation- sensitizing effects [53]. Canertinib has previously undergone phase II clinical trials for metastatic breast cancer (NCT00051051), although these studies were not specific to TNBC. Our model highly ranked Canertinib for the LAR subtype in both cell line- (2/100) and patient-derived data (19/100). Canertinib is also ranked in the Top 100 for the ML subtype patient-derived and cell line analyses (83/100 and 56/100, respectively), but is absent from the Top 100 for the BL1 subtype. MDA-MB-453 cells (LAR) were in fact significantly more responsive than either BT549 (ML) or HCC1937 (BL1) cells to the lowest dose of Canertinib tested (2.5 μM), but at higher concentrations the BL1 cells were just as responsive as LAR cells (Figure 5B). This may be due in part to the significant enrichment of Protein Kinase Inhibitors identified by GenEx-TNBC in the BL1 subtype (Figure 4).

### Bortezomib

Bortezomib is a proteasome inhibitor used to successfully treat multiple myeloma and mantle cell lymphoma [54] that is currently undergoing preclinical studies in TNBC and has been shown to enhance the efficacy of Fulvestrant in hormone receptor-positive metastatic breast cancer that is resistant to aromatase inhibitors [55]. GenEx-TNBC ranked Bortezomib as 17/100 for BL1 in the cell-derived analysis. It is also ranked 80/100 for patient-derived analysis of LAR. Cell growth assays confirm that the BL1 and LAR cell lines are significantly more responsive to Bortezomib than the ML cell line (Figure 5C).

### Tretinoin

Tretinoin, also known as all trans retinoic acid, has been used to treat acne [54] as well as acute promyelocytic leukemia [54]. In combination with doxorubicin and the histone deacetylase inhibitor entinostat, it has efficacy in MDA-MB-231 TNBC cells, representative of the MSL subtype [56] GenEx-TNBC ranked Tretinoin as the top drug for BL1 in our cell-line derived analysis. However, all 3 cell lines tested responded similarly to Tretinoin, with growth inhibition occurring only at the highest concentration tested and no statistically significant difference between cell lines (20 μM, Figure 5D).

### AMG900 efficacy in BL1 vs. BL2 TNBC cell lines

We next sought to investigate whether GenEx-TNBC could predict relative drug sensitivities *in vitro* between the two most highly related TNBC subtypes, BL1 and BL2. GenEx-TNBC ranked AMG900, a pan-Aurora kinase inhibitor, in the Top 100 for the BL1, but not BL2, subtype, where it is in the Top 500. Within the BL1 subtype, AMG900 is ranked 11/100 in patient- and 72/100 in cell line-derived analysis, and AMG900 targeted four BL1-specific differentially expressed gene products in our model, including G1/S-specific cyclin-E1 (CCNE1), a CDK-regulating protein, aurora kinase A (AURKA), aurora kinase B (AURKB), and the Myc proto-oncogene protein (MYC), a transcription factor that plays a key role in cell cycle progression.

**Figure 6:**
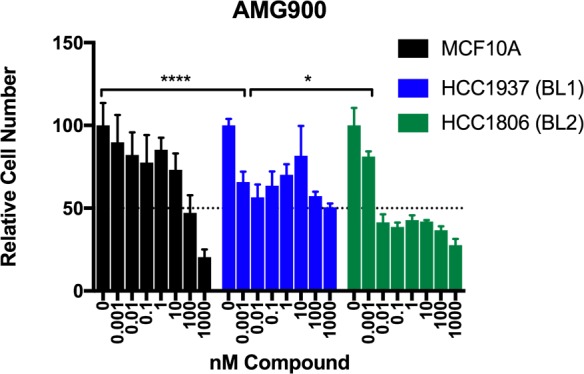
Testing of the pan Aurora kinase inhibitor AMG900 in basal-like TNBC cells Crystal violet staining of MCF10A (non-cancer), HCC1937 (BL1), and HCC1806 (BL2) cells grown in the presence of the indicated concentrations of AMG900 for seven (7) days. Data for each cell line were normalized to the appropriate DMSO control. Two-way ANOVA with Bonferroni *post hoc* multiple comparisons test (asterisks indicate per-dose comparison of HCC1806 or HCC1937 to MCF10A). Data are presented as the mean +/− standard deviation (S.D.) for a single experiment performed with 6 technical replicates that is representative of at least two independent experiments (biological replicates).

Given the differential rankings between the two basal-like subtypes, we predicted that AMG900 would be efficacious against these subtypes and further hypothesized that BL1 may be more sensitive to AMG900 than BL2. AMG900 has previously been studied in breast cancer cell lines [44], where its potency is associated with p53 dysfunction, a central feature of TNBC [57]. To validate our GenEx-TNBC prediction, we tested AMG900's effect on cell proliferation in HCC1806 (BL2 subtype, p53 null) and HCC1937 (BL1 subtype, p53 null and *BRCA1* mutant) TNBC cell lines versus the non-transformed mammary epithelial cell line MCF10A (Figure 6). At sub-nanomolar concentrations, both HCC1806 and HCC1937 cells are significantly more responsive to growth inhibition by AMG900 than MCF10A (two-way ANOVA Interaction p<0.0001 with *post hoc* Bonferroni multiple comparison).

Because AMG900 was similarly potent with respect to growth inhibition in the BL1 (HCC1937) and BL2 (HCC1806) cell lines at sub-nanomolar concentration, we asked whether there were differences in specific biological effector tiers predicted to be affected by AMG900 via our method. We found that for BL1, AMG900 was statistically significantly predicted to exert its effects at all four tiers (gene/protein, PPI, pathway and function) whereas for BL2, AMG900 had significant associations at only the PPI and function levels (Supplementary Table 4). These findings suggest that direct interaction of drugs with the relevant gene products (proteins) may be more likely to drive therapeutic benefit than targeting of higher-order processes such as cellular pathways, functions, and proteins that interact with disease-associated proteins. In comparing our biological testing and computational modeling results, we substantiate further evidence of previous findings [8] that drug sensitivity is not only conferred by increased expression of the drug's direct target, but also by the global cellular effects of that drug.

## MATERIALS AND METHODS

### Database preparation of experimentally validated drug-target signatures and higher-order biological associations

A master database of experimentally validated drug-protein associations, also referred to as drug-target signatures, was curated from the following publicly accessible databases: (1) Drug Gene Interaction Database (DGIdb) [23] (accessed April 20, 2016), and (2) Comparative Toxicogenomics Database (CTD) [24] (accessed March 4, 2016). Within the CTD, only interactions with a “binder” designation were used to establish associations. Also included were any known effects on protein targets, such as enzyme activation/inhibition, protein agonism/antagonism, and so forth. It is thought the combination of DGIdb and CTD, each containing extensively manually curated drug-gene interactions extracted from multiple other databases such as DrugBank [58], ChEMBL [59], PubChem [60], and others, serves a comprehensive dataset of interactions to date. This curation resulted in a total of 21,819 protein associations for 8,020 drugs.

From this master database of associations, sub-datasets were created to match the gene universe (e.g. set of all genes studied) of the microarray platform being used. This method has been previously described in our previous study Issa *et al* [61]. Thus, for each unique platform used in a study, the sub-dataset included only targets whose genes had probes in that microarray platform. This step is critical as proper statistical analysis can only be conducted if the gene universe encompassing the drug-target signatures and that of the disease signature under study are exactly the same.

Drugs were also associated with biological pathways and functions through their direct protein targets if an annotation existed. Annotations were retrieved from DAVID Functional Annotation Tool [21] for functions and ConsensusPathDB [22] for biological pathways. These collectively allowed for the most comprehensive and up to date annotation dataset to be used. Integrated pathway databases include BioCarta [62], Edinburgh Human Metabolic Network [63], HumanCyc [64], INOH [65], KEGG [66], PharmGKB [67], NCI Pathway Interaction Database [68], Reactome [69], SMPDB [70], and WikiPathways [71]. Functions were obtained from the Gene Ontology [72].

### Differential gene expression of TNBC subtype-specific genes and higher-order biological analytics

Patient-derived TNBC gene expression (RNAseq) data for primary tumors were obtained from The Cancer Genome Atlas (TCGA) [20] via extraction from the UCSC Cancer Genome Browser [73]. For each patient sample, the TNBCtype webtool [20] was used to classify that sample into the subtype according to Lehmann *et al* [4]. Differential gene expression analysis (DGEA) was performed in R [74] where one TNBC patient subtype group was compared to the others collectively. For example, if the subtype in question was BL1, then it was compared against BL2, IM, LAR, ML, and MSL as a single collective group (Figure 1). Up- and down-regulated genes with two-tailed t-test p-value < 0.05 were considered statistically significantly associated with the TNBC subtype in question. If the list of differential genes was very large, then only the top 1,500 up-regulated and top 1,500 down-regulated genes based on absolute fold change were chosen for further analysis as DAVID imposes a limitation on gene list size (maximum of 3000 genes). In the case of cell line samples, we obtained breast cancer cell line gene expression profiling data from Neve *et al* [19], assigning all TNBC cell lines to subtypes according to subtyping performed by Lehmann *et al* [4]. A single cell line was chosen to best represent each subtype, that which is most closely correlated in Lehmann *et al* with the subtype in question and also present in the Neve *et al* gene expression data. These were HCC1937 for BL1, SUM149PT for BL2, HCC1187 for IM, MDA-MB-453 for LAR, BT549 for ML, and HS578T for MSL. We calculated standard scores (z-values) for each gene being measured, comparing the expression for these individual cell lines to TNBC cell lines of all other subtypes as a group. The top 1,500 up-regulated and top 1,500 down-regulated genes based on z-value were utilized.

Protein-protein interactions (PPIs) for differentially expressed genes were obtained from the STRING database using a high confidence score cutoff of >0.7 [26]. Similar to the drug-target signature database, PPIs were filtered into sub-datasets according to the gene universe of the microarray platform in question. DAVID and ConsensusPathDB were also used to annotate pathways and functions for differentially expressed genes using P-value < 0.05 for discovery purposes.

### Causal analysis algorithm for directionality in comparing drug and disease networks

The statistical significance of drug-disease association was determined at the level of genes (direct protein targets), PPIs, pathways, and functions. Lamb *et al* previously demonstrated using cancer cell lines that drugs causing gene expression signatures that are inversely correlated to the gene expression signature of the disease state were likely to be therapeutic [16]. In GenEx-TNBC, inverse associations between specific drug actions and TNBC subtype-related biological mediators are obtained by quantifying the ability of the drug to: (1) inhibit the activated biological mediators, and (2) activate the inhibited mediators in a given TNBC subtype. Drugs with the potentiality to perform both tasks simultaneously and to the greatest extent are considered to have the greatest therapeutic potential and prioritized for testing.

In calculating the inverse statistical significance between drug and TNBC subtype at each level of biological activity, activating drug interactions were considered a “hit” when matched with down-regulated genes, while deactivating drug interactions were considered a “hit” when matched with up-regulated genes. Unknown interactions in terms of directionality were considered a “hit” when the gene was significantly regulated in either direction, thus providing an agnostic term. A schematic of this approach is shown in Figure 2A. This theme was carried through to the PPI, pathway and function levels.

The drug-disease network matching process is mathematically represented in Equations 1-6 corresponding to each biological tier:

$$N_{g}\left(i,k\right)=\left|\;{\hat{X}}_{i}\cap {\hat{D}}_{k}\right|+\left|\;{\overset{}{\hat{X}}}_{i}\cap {\overset{}{\hat{D}}}_{k}\right|+\left|\;{\bar{X}}_{i}\cap \left({\hat{D}}_{k}\cup {\overset{}{\hat{D}}}_{k}\right)\;\right|$$ (1)

$$N_{p}\left(i,k\right)=\left|\;{\hat{X}}_{i}\cap {\hat{D}}_{k}\right|+\left|\;{\overset{}{\hat{X}}}_{i}\cap {\overset{}{\hat{D}}}_{k}\right|+\left|\;{\bar{X}}_{i}\cap \left({\hat{D}}_{k}\cup {\overset{}{\hat{D}}}_{k}\right)\;\right|$$ (2)

$$N_{f}\left(i,k\right)=\left|\;{\hat{X}}_{i}\cap {\hat{D}}_{k}\right|+\left|\;{\overset{}{\hat{X}}}_{i}\cap {\overset{}{\hat{D}}}_{k}\right|+\left|\;{\bar{X}}_{i}\cap \left({\hat{D}}_{k}\cup {\overset{}{\hat{D}}}_{k}\right)\;\right|$$ (3)

$$P\left(i,k\right)=\;\left|\left({\hat{X}}_{i}\cup {\overset{}{\hat{X}}}_{i}\cup {\bar{X}}_{i}\right)\cap \left({\hat{D}}_{y}\cup {\overset{}{\hat{D}}}_{y}\right)\;\right|$$ (4)

where *N_g_*(*i,k*), *N_p_*(*i,k*), *N_f_*  (*i,k*), and *P*(*i,k*) are functions representing the total number of *k* gene products, pathways, functions, and PPIs targeted by drug *i*, respectively, with respect to a given TNBC subtype. ${\hat{X}}_{i}$ represents the set of all agonistic drug-gene product, pathway and function interactions, and ${\hat{D}}_{k}$ represents the set of all down-regulated gene products, pathways, and functions as it pertains to the relevant function *N*. $\left|\;{\hat{X}}_{i}\cap^{\text{​}}{\hat{D}}_{k}\right|$ represents the cardinality of the intersection between sets ${\hat{X}}_{i}$ and ${\hat{D}}_{k}$, thus quantifying the total number of biological elements, with respect to function *N*, that are down-regulated in the TNBC subtype and could have an agonistic relationship with drug *i*. Similarly, ${\hat{X}}_{i}$ and ${\hat{D}}_{k}$ represent the set of all antagonistic drug-biological mediator interactions and the set of all up-regulated biological mediators of the relevant function *N*. $\left|\;{\hat{X}}_{i}\cap^{\text{​}}{\hat{D}}_{k}\right|$ is the cardinality of the intersection between sets ${\hat{X}}_{i}$ and ${\hat{D}}_{k}$ representing the total number of biological elements up-regulated in the TNBC subtype that could have an antagonistic relationship with drug *i*. As many drug associations obtained from the available databases do not indicate directionality, which is represented by the set ${\bar{X}}_{i}$, a direction-agnostic term was also included. Thus, $\left|\;{\bar{X}}_{i}\cap \left({\hat{D}}_{k}\cup {\overset{}{\hat{D}}}_{k}\right)\;\right|$ is the cardinality of the intersection of all drug-biological mediator direction-agnostic interactions with the union of all down- and up-regulated biological elements. With respect to protein-protein interactions, information was extracted as agnostic to directionality as well. Thus, *P*(*i, k*) could only be expressed as an agnostic term. $\left|\left({\hat{X}}_{i}\cup {\hat{X}}_{i}\cup {\bar{X}}_{i}\right)\cap \left({\hat{D}}_{y}\cup {\hat{D}}_{y}\right)\;\right|$ therefore represents the cardinality of the intersection between all drug-biological mediator interactions (the union of agonistic, antagonistic and agnostic interactions) and all proteins interacting with all gene products associated with the TNBC subtype in question (the union of down- and up-regulated gene products).

To determine whether a statistically significant association existed between each drug and the TNBC subtype at each biological tier, the hypergeometric test was performed in R (Figure 2B). Input values for the hypergeometric test were obtained from each function *N* noted above. The hypergeometric test is mathematically represented by the following equation:

$$P_{i}\left(N=x\right)=\frac{\left(\begin{array}{c} \gamma \\ x \end{array}\right)\left(\begin{array}{c} n-\gamma \\ \delta -x \end{array}\right)}{\left(\begin{array}{c} n\\ \delta \end{array}\right)}$$ (5)

where, for each tier, *P_i_(N = x)* represents the hypergeometric test function for drug *i*, *n* is the total number biological elements in the given universe space, δ is the total number of TNBC subtype-associated biological elements, γ is the total number of drug-biological element interactions, and *x* is the number of hits for drug *i* as obtained from the relevant function *N* noted above. Drugs with p≤0.05 had their P-values log-transformed then normalized against the value of the most significantly-associated drug, resulting in values on the 0-1 unit range. All non-significant P-values (p>0.05) were normalized to the value of 0.

Lastly, each drug's Z-score (*Z_i_*), which represents the final quantification of the drug-TNBC subtype association, is calculated for ranking using the following equation:

$$Z_{i}=\text{aA}+\text{bB}+\text{cC}+\text{dD}$$ (6)

where *A*, *B*, *C*, and *D* correspond to the normalized hypergeometric test P-values for drug-gene product, −pathway, −function, and –PPI associations, respectively. *a*, *b*, *c*, and *d* represent coefficient values of 2, 1, 1, and 1 with respect to each biological tier. As previously described in Issa *et al* [59], coefficient values were determined to best prioritize direct drug-gene product interactions over indirect interactions at higher-order biological tiers while also allowing for the prioritization of drugs that do not necessarily have direct interactions but may be therapeutic through indirect mechanisms. Using the final calculated Z-score, drugs are ranked in descending order (e.g. the drug with the highest Z-score is considered the number one top-ranked drug for a particular TNBC subtype). Thus, a high Z-score indicates a drug's polypharmacological and multi-tiered potential to serve as a therapeutic for a given TNBC subtype.

### Cell culture

The Lombardi Comprehensive Cancer Center (LCCC) Tissue Culture Shared Resource provided MCF10A non-transformed mammary epithelial cells as well as MDA-MB-231, BT549 and HCC1937 breast cancer cells. HCC1806 breast cancer cells were purchased from ATCC (Manassas, VA). MDA-MB-453 cells were kindly provided by Dr. Anna Riegel (LCCC). Cells routinely tested negative for *Mycoplasma spp*. contamination, and were authenticated by short tandem repeat (STR) profiling for 9 standard loci and Y chromosome-specific amelogenin by the LCCC Tissue Culture Shared Resource to verify their authenticity, most recently in March 2017. Cells were maintained in a humidified incubator with 95% air: 5% carbon dioxide. HCC1806, HCC1937, MDA-MB-453 and MDA-MB-231 cells were grown in improved minimal essential media (IMEM; Life Technologies, Grand Island, NY) supplemented with 10% heat-inactivated fetal bovine serum (FBS, purchased from the LCCC Tissue Culture Shared Resource). BT549 cells were grown in improved minimal essential media (IMEM; Life Technologies, Grand Island, NY) supplemented with 0.023 IU/mL insulin (Life Technologies) and 10% FBS. MCF10A cells were grown in a 1:1 mixture of Ham's F12: Dulbecco's modified essential media (DMEM, Life Technologies) supplemented with 20 ng/ml epidermal growth factor (EGF), 10 μg/ml insulin, 0.5 μg/ml hydrocortisone, 100 ng/ml cholera toxin, and 5% horse serum (purchased from either the LCCC Tissue Culture Shared Resource or Sigma Aldrich, St. Louis, MO).

### Compounds

Mecamylamine (Sigma Aldrich), Canertinib (AK Scientific, Union City, CA), Bortezomib (AK Scientific), Tretinoin (AK Scientific), and AMG900 (Selleckchem, Houston, TX) were resuspended in dimethyl sulfoxide (DMSO – Canertinib, Bortezomib, Tretinoin, AMG900) or 200 proof ethanol (EtOH – Mecamylamine) at a concentration of 10 mM, stored at -20°C, and used at the indicated concentrations.

### Cell proliferation assays

Cells were seeded into 96-well plastic tissue culture dishes at 2,000 (MCF10A and HCC1806), 5,000 (MDA-MB-453 and HCC1937), or 7,500 (BT549) cells per well on day 0. On day 1, each plate was treated with a range of concentrations of the following compounds and the appropriate solvent control: CI-1033 (Canertinib), Tretinoin, Bortezomib, or Mecamylamine (2.5 to 20 uM); or AMG900 (1 pM to 1000 nM). Plates were re-dosed on day 3 or 4 and stained on day 7. Prior to staining, each plate was rinsed once with 1X Phosphate-Buffered Saline (PBS), and MDA-MB-453 and BT549 cells were first fixed with 50 μl/well of 3.2% paraformaldehyde (Electron Microscopy Services/VWR, Radnor, PA) for 5 minutes at room temperature. To stain, plates were incubated with 100 μl/well of a solution of 0.5% w/v crystal violet (Sigma Aldrich) dissolved in 25% methanol: 75% water at 4°C for 15 minutes. Excess stain was removed and each plate was washed 5-6 times with deionized H_2_O and allowed to air dry completely. Stained cells were rehydrated in a 0.1M sodium citrate buffer dissolved in 50% ethanol: 50% water, then read on a plate reader at an absorbance of 550nm. Each assay included 6 technical replicates, and was performed twice independently (biological duplicates).

### Image and statistical analyses

All statistical analyses were performed in Prism 6.0 or 7.0 (Graphpad, San Diego, CA), and are specified in the figure legends. For cell proliferation assays, non-linear regression analyses were performed using log[inhibitor] vs. normalized response, or, log[inhibitor] vs. normalized response - variable slope, parameters. A comparison of fits was used to determine the preferred analysis model, and curve fits with an R^2^ value ≥ 0.8 are shown. All data are presented as the mean ± standard deviation (S.D.). Two-way analysis of variance (ANOVA) with post hoc Tukey's multiple comparisons test was used to determine differences between cell lines at individual doses. Statistical significance is defined as a *P* value of ≤0.05. ^*^p≤0.05, ^*^p≤0.01, ^***^p≤0.001, ^****^p≤0.0001.

## CONCLUSIONS

TNBC is a devastating disease with poor survival outcomes, increasing costs, and a relatively small therapeutic armamentarium. Transcriptomics and proteomics have pointed toward TNBC as a heterogeneous sub-group of breast cancer where multiple phenotypically distinct subtypes exist. As hormone receptor positivity and PAM50 gene expression signatures [75] affect treatment selection, TNBC subtyping should also be embraced for drug discovery. Furthermore, like other tumors, TNBC is variable in having actionable gene mutations or other clear therapeutic targets, which further contributes to its complexity. Here we have devised a novel, multi-tiered drug discovery and repurposing platform entitled GenEx-TNBC inspired by the concept of disease-therapy inverse associations. Through this first-in-class platform, TNBC subtype-directed drugs are prioritized by using a holistic approach targeting all subtype-specific biological elements at different tiers, including direct gene products (proteins), protein-protein interactions, pathways, and molecular functions. This circumvents traditional limitations imposed by prioritization of single actionable targets.

We demonstrated that our subtype-specific TNBC biological network models reflect mechanisms that have been previously attributed to those subtypes in the scientific literature. This validates our strategy of inter-subtype comparisons in differential gene expression rather than a conventional “cancer versus normal” approach, and highlights the potential of GenEx-TNBC to propose alternative subtype-specific mechanisms that have not been recognized for targetability. Furthermore, we prioritized drugs against each subtype based on network coincidence at multiple scales of biological action. GenEx-TNBC prioritized drugs and drug classes that were confirmed or being explored for effectiveness against a given subtype in the literature, as well as drugs that have not been evaluated against TNBC and are potential candidates for subtype-specific assessment. We tested four GenEx-TNBC predictions in cell lines corresponding to specific subtypes, and validated several of them, the most interesting of these being the susceptibility of the LAR subtype of TNBC to an nAChR antagonist (Mecamylamine) and a pan-ErbB tyrosine kinase inhibitor (Canertinib). These data support further testing and validation of other novel agents predicted by GenEx-TNBC which may be successfully repurposed for TNBC such as etodolac, a non-steroidal cyclooxygenase inhibitor used as an anti-inflammatory agent predicted to target the ML subtype. Etodolac and other drugs of this class could present repurposing opportunities to the ML subtype of TNBC. The drug prioritization scheme of GenEx-TNBC does not depend on association with the subtype at all four assessed levels of activity, but instead can rely on a mixture of one or more levels. In applying this concept, GenEx-TNBC was successful in predicting well-known drugs for TNBC subtypes as well as discriminating a drug's potency across subtypes.

As GenEx-TNBC is a polypharmacology-based approach rooted in systems biology, it can be adapted for any other disease state where gene expression signatures exist. Breast cancer is a well-established case in which molecular profiling has matured to a point where it is a key component of clinical practice that impacts treatment decisions. It is now appreciated that other malignancies, such as colorectal cancer [76], glioblastoma [77], and pancreatic cancer [78], can be classified into two or more subtypes. These subtypes have prognostic value, but this has not yet translated to widespread adoption of targeted therapies to address the specific molecular features of a tumor belonging to a particular subtype. Implementation of strategies like GenEx-TNBC in these contexts could be very impactful.

## SUPPLEMENTARY MATERIALS FIGURES AND TABLES











## Abbreviations

ATRA: All-Trans Retinoic Acid
AURKA: Aurora Kinase A
AURKB: Aurora Kinase B
BL1: Basal-like 1
BL2: Basal-like 2
CCNE1: G1/S-Specific Cyclin-E1
CMap: Connectivity Map
CTD: Comparative Toxicogenomics Database
CTLA-4: Cytotoxic T-Lymphocyte-Associated Protein 4
DGEA: Differential Gene Expression Analysis
DGIdb: Drug Gene Interaction Database
DMEM: Dulbecco's Modified Essential Media
EGF: Epidermal Growth Factor
EGFR: Epidermal Growth Factor Receptor
EMT: Epithelial–Mesenchymal Transition
ER+: Estrogen Receptor-Positive
FGFR: Fibroblast Growth Factor Receptor
IM: Immunomodulatory
LAR: Luminal Androgen Receptor
LCCC: Lombardi Comprehensive Cancer Center
ML: Mesenchymal-like
MSL: Mesenchymal Stem-like
PARP: Poly(ADP-Ribose) Polymerase
PBS: Phosphate-Buffered Saline
PD-1: Programmed Cell Death Protein 1
PDGFR: Platelet-Derived Growth Factor Receptor
PPI: Protein-Protein Interaction
RXR: Retinoid X Receptor
TCGA: The Cancer Genome Atlas
TFG-β: Transforming Growth Factor Beta
TNBC: Triple Negative Breast Cancer
VEGFR: Vascular Endothelial Growth Factor Receptor
