# Supplementary material for: Differential prioritization of therapies to subtypes of triple negative breast cancer using a systems medicine method
Source: Oncotarget. 2017 Oct 9;8(54):92926–42. doi: 10.18632/oncotarget.21669 (PMC5696233; doi:10.18632/oncotarget.21669)
Supplement: Supplementary file 1 [file oncotarget-08-92926-s001.pdf]

Differential prioritization of therapies to subtypes of triple negative breast cancer using a systems medicine method

SUPPLEMENTARY MATERIALS

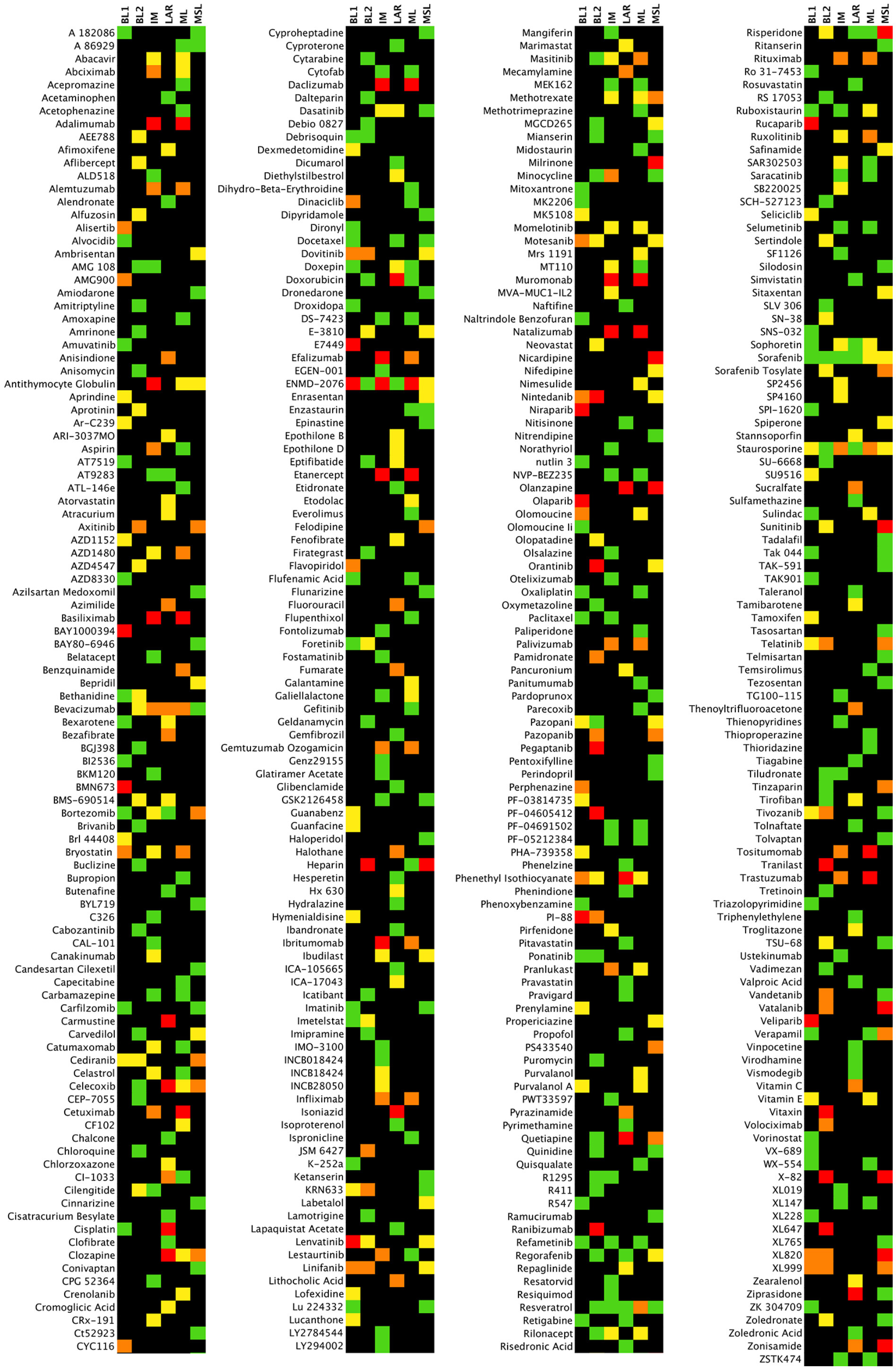

Supplementary Figure 1: Heat map representation of GenEx-TNBC drug rank lists for TNBC subtypes characterized by patient-based gene expression data. Distribution of drugs prioritized in the top 100 from rank lists generated for each TNBC subtype. Red, orange, yellow, and green squares represent the placement of the drug in the top 10, 25, 50, and 100, respectively. Black squares indicate that the drug was not ranked in the top 100.

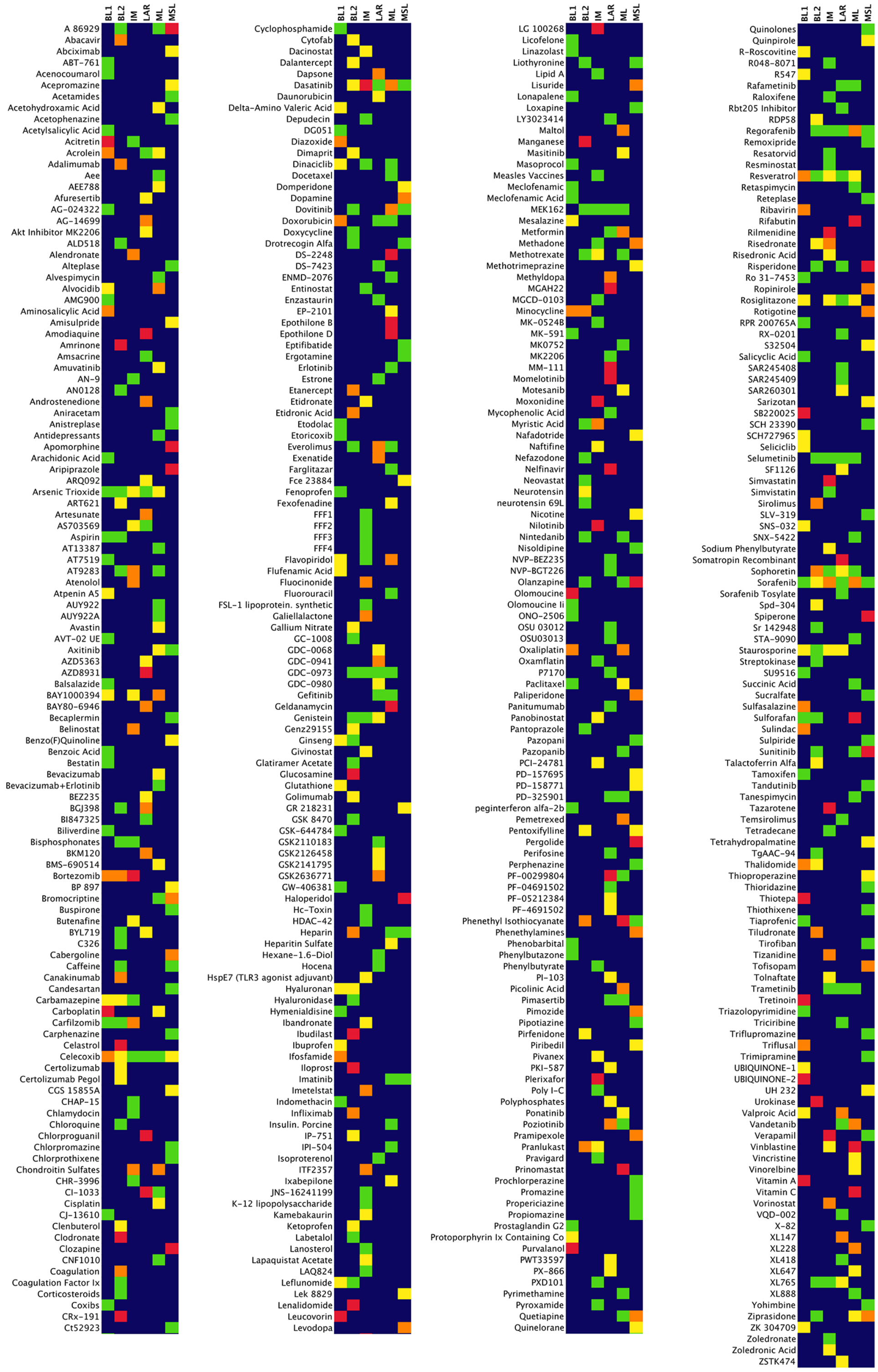

Supplementary Figure 2: Heat map representation of GenEx-TNBC drug rank lists for TNBC subtypes characterized by cell line-based gene expression data. Distribution of drugs prioritized in the top 100 from rank lists generated for each TNBC subtype. Red, orange, yellow, and green squares represent the placement of the drug in the top 10, 25, 50, and 100, respectively. Blue squares indicate that the drug was not ranked in the top 100.

Supplementary Table 1: Top 100 drugs ranked by TNBC subtype association score for patient-based and cell line-based analyses

See Supplementary File 1

Supplementary Table 2: Literature-based validations of drugs ranked in the top 50 of each TNBC subtype in the case of patient-derived disease network modeling

See Supplementary File 2

Supplementary Table 3: Pathways significantly overrepresented in up- and down-regulated genes in patient-based models of TNBC subtypes (using ConsensusPathDB [23]) for P-value <.05. For simplicity, only resulting pathways from the KEGG database are included.

See Supplementary File 3

Supplementary Table 4: Statistical significance of coincidence with multiple scales of biological action in each TNBC subtype for the drug AMG900, looking at both patient-based and cell-line based disease networks

See Supplementary File 4
